# Persistent Cellular Motion Control and Trapping Using Mechanotactic Signaling

**DOI:** 10.1371/journal.pone.0105406

**Published:** 2014-09-10

**Authors:** Xiaoying Zhu, Roland Bouffanais, Dick K. P. Yue

**Affiliations:** 1 Singapore University of Technology and Design, Singapore, Singapore; 2 Department of Mechanical Engineering, Massachusetts Institute of Technology, Cambridge, Massachusetts, United States of America; Massachusetts Institute Of Technology, United States of America

## Abstract

Chemotactic signaling and the associated directed cell migration have been extensively studied owing to their importance in emergent processes of cellular aggregation. In contrast, mechanotactic signaling has been relatively overlooked despite its potential for unique ways to artificially signal cells with the aim to effectively gain control over their motile behavior. The possibility of mimicking cellular mechanotactic signals offers a fascinating novel strategy to achieve targeted cell delivery for *in vitro* tissue growth if proven to be effective with mammalian cells. Using (i) optimal level of extracellular calcium ([Ca^2+^ ]_ext_ 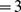 mM) we found, (ii) controllable fluid shear stress of low magnitude (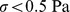), and (iii) the ability to swiftly reverse flow direction (within one second), we are able to successfully signal *Dictyostelium discoideum* amoebae and trigger migratory responses with heretofore unreported control and precision. Specifically, we are able to systematically determine the mechanical input signal required to achieve any predetermined sequences of steps including straightforward motion, reversal and trapping. The mechanotactic cellular trapping is achieved for the first time and is associated with a stalling frequency of 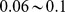 Hz for a reversing direction mechanostimulus, above which the cells are effectively trapped while maintaining a high level of directional sensing. The value of this frequency is very close to the stalling frequency recently reported for chemotactic cell trapping [Meier B, et al. (2011) Proc Natl Acad Sci USA 108:11417–11422], suggesting that the limiting factor may be the slowness of the internal chemically-based motility apparatus.

## Introduction

One of the remarkable things about many eukaryotic cells is how effective they are at sensing minute levels of mechanical stimulation, while living in a constantly changing biomechanical environment. Mechanosensation is a widespread phenomenon in a host of different single-celled and multicellular organisms [1].

Recent studies indicate that mechanical forces have a far greater impact and a more pervasive role on cell functions and fate than previously thought [1]. There is now mounting evidence that eukaryotic cells such as cancer cells, fibroblasts, endothelial cells, amoebae and neutrophils migrate directionally following a complex biophysical response elicited by the exquisite mechanosensitivity of these cells to shear flows [2]–[7]. Directional cell motility is ubiquitous in both normal and pathophysiological processes [3], [4]. From the medical standpoint, mechanotactic signaling and its induced directional cell migration play a key role in the immune system and metastasis responses and spreading [8], [9]. From a developmental biology standpoint, the directional rearrangement of cells induced by fields of external stimuli is a key mechanism involved in metazoan morphogenesis; more specifically in early embryonic development: gastrulation followed by organogenesis [10].

Chemotactic signaling and the associated directional migration have received tremendous attention in the past decades. In comparison, mechanotactic signaling has been relatively less studied, though its importance has proved to be central in a series of recent experiments involving eukaryotic cells [2]–[7]. Mechanotaxis encompasses several different responses due to various mechanostimuli: e.g. substrate stiffness for durotaxis [2], flow shear stress [5], pressure for osmotaxis, etc. From the medical standpoint, mechanotactic signaling is responsible for regulating leukocyte functions, e.g., increasing motility and phagocytic capabilities [11]. Furthermore, mechanotaxis has recently been considered as a way to control and manipulate cell motility [12], [13], which could potentially lead to innovative applications in biotechnology and, more precisely, in tissue engineering [14], [15] if proven to be effective with mammalian cells. However, no cellular motion controller based on mechanotactic signaling has ever been reported to yield the accurate, persistent and reversible control over cell motion—including cellular immobilization—required by practitioners. The commonly employed strategy to manipulate cells consists in imposing specific environmental cues. The classical way of setting those cues is primarily relying on biochemical functionalities, including chemotaxis. It is believed that *shearotaxis*—defined as the cellular response of directional migration elicited by the mechanosensing of fluid shear stress; mechanotaxis and shearotaxis shall be used interchangeably in the sequel—could be used to improve the precision and robustness of targeted cell delivery. These demonstrate the need to establish the existence of a mapping between a desired predetermined cellular motile output on the one hand, and the experimental inputs and controls of the environment on the other hand.

A large body of experimental evidences have shown that directional cell migration can be achieved through cellular sensing of chemical gradients. To generate chemotactic signals, microfluidic setups are commonly employed with intricate designs aimed at producing specific local gradients of the concentration of the chemical factor associated with the particular type of cells considered. However, such designs require a very careful calibration of the chemical gradient. On the contrary, mechanotactic signaling using fluid shear stress offers an alternative robust means of performing directed cellular guiding as is shown in our study. In this work, we show that by mimicking a mechanotactic signal, a dynamic and persistent control of cell motility is successfully achieved, including fast course reversals and cellular trapping. To that aim we follow the general approach of assessing the shearotactic controllability of amoeboid migrating cells with systematic and controlled measurements, and then identifying the key control and environmental parameters and their respective influences on cellular guiding and trapping.

We follow the general approach of Décavé *et al.* [5] and Fache *et al.* [17], in which the amoeboid model system *Dictyostelium discoideum* (Dd) is employed within a microfluidic cell system that allows the application of stable temporally-controlled shear stresses mimicking mechanotactic signaling. This system permits direct visualization of transient responses of multiply seeded cells and the swift reversal of the flow within one second, thereby almost instantly changing the stimulus direction. Within this well-controlled *in vitro* environment, multiple independent single-cell tracking can simultaneously be achieved, allowing us to obtain quantitative statistical characterizations of the directed migration. The key differences between the present study and the seminal works by Décavé et al. [5] and Fache et al. [17] lie in our search for and selection of the values for the two prominent controlling parameters—shear stress level and extracellular calcium concentration. Specifically, we find that using very low mechanotactic signaling shear stresses (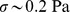) and an “optimal” calcium concentration of 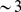 mM, substantially enhanced cell migratory responses, in terms of both speed and directionality, are obtained compared to prior studies [5], [17]. Because of these differences in levels of shear stresses and extracellular calcium, we were able to achieve, for the first time, mechanotactic cell trapping. It is noteworthy that these levels of shear stress and calcium concentration are close to those encountered in the cell's natural environment. Our particular interest and focus in such low shear stress levels stem primarily from two important facts: first, the known ability of cells to be driven *in vivo* by very low shearotactic stimuli—e.g. interstitial flows [3], and second, the sharp reduction in cell detachment from the surface for this range of shearostimuli. Indeed, preventing flow-induced cell detachment is instrumental as the latter entirely suppresses all control on cell migration [18].

Our results establish the existence of a range of optimal values for the shear stress as well as clear evidences of an optimal value for the extracellular calcium concentration that enable persistent directionality in shearotactic cell guiding. Using the found appropriate levels of shear stress and extracellular calcium, we succeeded in appropriately signaling to the cells, thereby leading to any prescripted one-dimensional migrating path involving sequences of straightforward motions, immobilizations and course reversals in any order. We investigated each of these three motility components in terms of the obtained responses relative to the prescribed mechanotactic signal—the associated molecular details are beyond the scope of this study. Cellular course reversal is obtained by abruptly reversing the shear flow direction. Immobilization is attained by stalling and trapping the cell using a directional switching of the prescribed shear flow at relatively high frequency. More precisely, when the switching rate is above a stalling frequency of 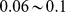 Hz, shearotactic cell trapping is achieved. It is remarkable that this value is very close to the stalling frequency recently reported for chemotactic cell trapping [19], thereby suggesting that, under such conditions, the internal chemically-based apparatus may be the limiting factor for cell motility.

## Results

### Prescripted Cellular Courses

**Figure 1:**
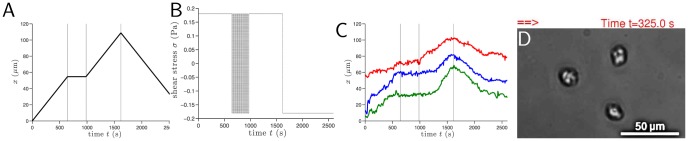
Prescripted cellular course. (A) Desired cellular output; (B) Imposed externally controlled mechanotactic signal; (C) Measured cellular displacement in the 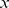 direction for 3 distinct cells with a different color for each cell. The observed small differences in cellular response between the three cells are rooted in the biological variability of this sample. The initial 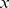 positions of the 3 cells have been arbitrarily shifted to circumvent the overlapping of the plots; (D) Instantaneous snapshot of the 3 cells in the observation area at instant 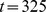 s. The double red arrow indicates the mechanostimulus direction. See SI Movie S1 for the complete migration of these 3 cells. The calcium concentration is 3 mM and the complete duration of the cellular course is 2600 seconds or 43 minutes. Cells are crawling over a plastic hydrophobic surface.

The possibility of mimicking cellular mechanotactic signals offers a fascinating novel strategy to achieve targeted cell delivery, which is key to growing tissues *in vitro*. Here, we show that with our microfluidic setup (see Materials & Methods), we successfully signaled cells through fluid shear stress. Specifically, using our found optimal cellular control conditions described below—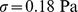, [Ca^2+^  ]_ext_ 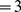 mM, we succeeded in imposing several prescripted one-dimensional migrating paths involving sequences of straightforward motions, immobilizations and course reversals in any order. From the practical motion-control standpoint, we were able to systematically map any desired cellular output onto the specifics of the external experimental inputs (Fig. 1 and Figs. S4(a) and S4(b)). Three particular prescripted cellular courses were sought corresponding to the following sequences of motion: (1) right, trapped, right, left; (2) right, trapped, left; (3) right, trapped, left, right (SI Movies S1, S2 and S3 respectively, and Fig. S4). It is worth adding that in all our experiments, the observed responses fell in the firm-adhesion regime of amoeboid crawling or blebbing; no rolling-adhesion regime—as commonly observed with leukocytes [20]—has been encountered.

### Influence of Shear Stress on Shearotactic Cell Guiding

From the perspective of accurate cell control, the intensity of the signal 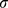 is bounded; the upper bound being reached when cells detach from the substrate. Beyond this value, the cells are washed away passively by the creeping flow and no longer respond actively to the mechanical signal (Table S1). Our results show that even for such a low value of 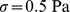, approximately 1 in 2 cells stop adhering to the substrate over the course of a 20-minute cellular guiding. However, by slightly reducing the magnitude of the shear stress, below 0.3 Pa, the detachment rate becomes marginal. As a consequence, our study focuses on very mild shear flows to ensure the persistence of controlled cellular guided migration. Note that the extracellular calcium level has very little influence on the detachment rate for low shear stress in the 0.2 Pa range.

**Figure 2:**
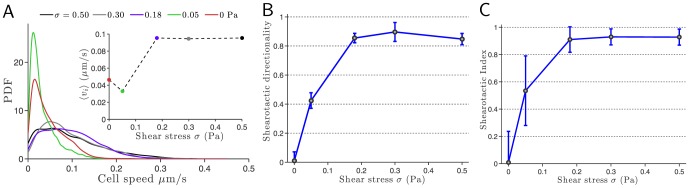
Influence of shear stress on shearotactic cell migration. (A) Influence on cell speed: probability density function (PDF) of the cell speed for five different magnitudes of the shearotactic signal with the associated average speed vs. shear stress in insert. For shear stress levels lower than 0.05 Pa, the average cell speed is of the order of the cell speed in the absence of any signal (insert). For values of 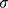 in the range 0.18 to 0.5 Pa, the average cell speed is found to be almost constant. This conclusion, established on the sole basis of first-order statistics of cell speed, is readily generalized by noticing the similarities in the PDFs of cell speed for 0.05 Pa on the one hand, and for 0.18 Pa on the other hand. (B) Evolution of the shearotactic directionality with the shear stress. (C) Evolution of the 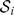 with the shear stress. 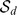 is a measure of the instantaneous ability of the cell to migrate in the signal's direction, while 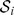 is an integrated measure of 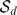 throughout the entire cell path. This subtle relationship between 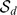 and 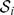 is reflected in the above variations with the shear stress where a plateau about 0.9 is reached for 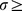
0.18 Pa. For all values of 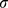, only cells adhering to the substrate throughout the entire duration of the experiment were considered and analyzed. All experiments were conducted with a soluble concentration of calcium, [Ca^2+^]_ext_, set and fixed at 3 mM and cells are crawling over a plastic hydrophobic surface.

Shear stress is known to influence both cellular speed and directionality (Fig. S1). Concerning the speed, we found that driving a cell with a signal as low as 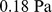 is an optimal shear stress level given the inherent trade-off between maximum average speed and minimum detachment rate (Fig. 2(a) and Table S1). Similarly to the study of other taxis, our measure of the shearotactic efficiency comprises two components: (i) the shearotactic directionality (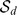) of the cells measured by 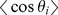, 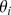 being the angle between the instantaneous cell velocity 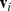 and the direction of the shear flow, arbitrarily chosen as the positive 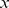-direction, and (ii) the shearotactic index (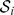), defined as the ratio of the distance traveled in the direction of the flow to the total length of the cell migration path during the same period. Cells moving randomly have a shearotactic directionality of 0, while cells moving straight along the flow have a directionality of 1; cells moving straight against the flow have a directionality of 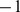. We find that the shear stress affects conspicuously both 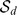 and 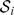 even at such mild levels; the lowest shear stress for which 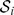 and 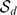 are near their maximum plateau level is 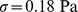 (Figs. 2(b) and 2(c)). In conclusion, shearotactically driving a cell at 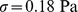 provides the best trade-off point between high speed and high shearotactic efficiency on the one hand, and low detachment rate on the other hand.

### Influence of the Extracellular Calcium Concentration

**Figure 3:**
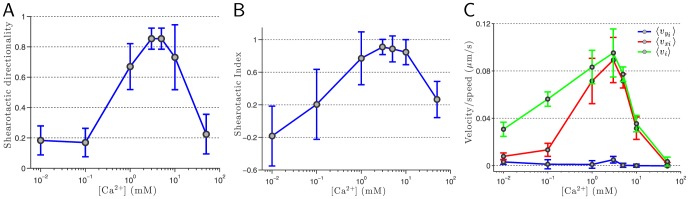
Influence of the extracellular calcium concentration on the shearotactic cellular response. (A) 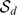. (B) 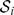. (C) Average cell speed 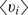, average 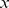-component (resp. 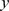-component) of the cell velocity 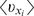 (resp. 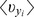) for a shearotactic signal pointing toward the positive 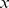 direction. At both ends of the calcium concentration range considered, the shearotactic efficiency is extremely poor as attested by the values of 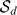 and 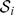. A high shearotactic efficiency is achieved for calcium concentrations in the 1–3 mM range. For the speed, a clear maximum is attained for a concentration of 3 mM. The optimal shear stress level of 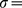
0.18 Pa is considered for the seven different values of the external calcium concentration. A log-scale is used for the calcium concentration on the 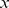-axis and for each value of [Ca^2+^]_ext_ the averaging process is based on a population comprising between 60 to 135 individual tracked cells for a duration of 1,200 seconds and with a sampling time of 15 seconds. Cells are crawling over a plastic hydrophobic surface.

Our study considers a wide range of extracellular calcium concentration from 10 µM to 50 mM. Our results prove the existence of a clear optimal value for the calcium concentration with regards to the average cell speed as well as for its directionality (Fig. 3). When subjected to a signal of magnitude 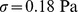, this optimal value is [Ca^2+^ ]_ext_ 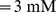 (Fig. 3). Not surprisingly, this optimal value is very close to the concentration of Ca^2+^ commonly found in soil solutions [21]. Our results are remarkable for two reasons. First, it has been reported that speed and directionality of cell movement are independently regulated and controlled [17]. However, such similar variations with Ca^2+^, and the exact same optimal value for the average cell speed and the shearotactic efficiency (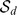 and 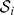) seem to contradict the independent regulation of speed and directionality. Second, a too high calcium concentration, [Ca^2+^ ]_ext_ 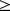
50 mM, actually suppresses almost completely the cellular motion as 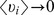 (Fig. 3(c)). To our knowledge, the existence of such an optimal calcium concentration for a given shear stress level, as well as the almost complete disappearance of shearotactic cellular guiding at relatively high calcium concentrations, [Ca^2+^ ]_ext_ 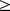
50 mM, have never been identified previously for vegetative Dd cells.

### One-dimensional Cell Motion Control

**Figure 4:**
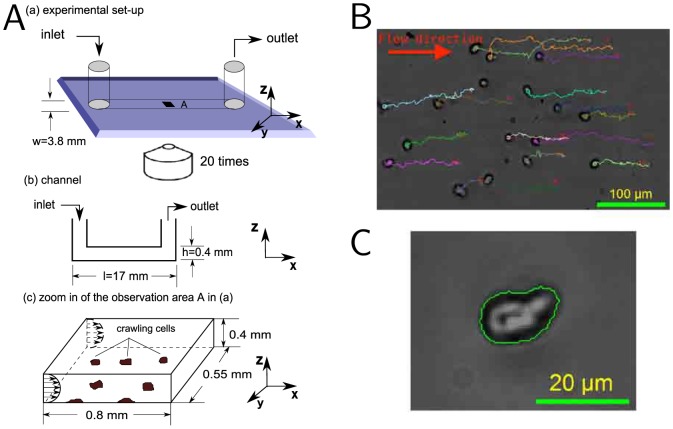
Cell motility device for investigating the effects of shearotactic signals on cell migration. (A) Schematic of the microfluidic device. The external flow circuit comprising the syringe pump—temporally controlling the shearotactic signal—is not represented but is connected to the inlet and outlet of the microfluidic channel. (B) Multiply seeded vegetative unpolarized Dd cells in their initial position with their respective single-cell centroid trajectories superimposed; the flow is towards the right with a shear stress of 0.18 Pa and a soluble calcium concentration of 3 mM. (C) Close-up of a single vegetative Dd cell with its detected boundary used to determine the cell's centroid. For each shear stress levels and calcium concentrations considered, a large number of cells are analyzed (Fig. S1), providing statistically relevant data.

Having identified the optimal values of the calcium concentration and of the shear stress level, we turn to the actual control of persistent directed motion. The first essential element in one-dimensional cell control is the ability to drive a single cell, or a group of cells, to steadily migrate in the same direction—the positive *x*-direction in our case with 205 different cells. Imposing a calcium concentration of 3 mM, we mechanically signal the cells with 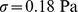, and we report several aspects of the cell kinematics characterizing its motion. To accurately track the Lagrangian path of a cell in the microchannel (Fig. 4(a)), we used a reduced sampling time, 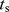, of 3.5 seconds which was found appropriate given the average cell speed of approximately 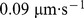 reported in Fig. 3(c). At this average speed, the cellular displacement is approximately 2% of the cell size which falls into the range of detectable displacements of our tracking system.

**Figure 5:**
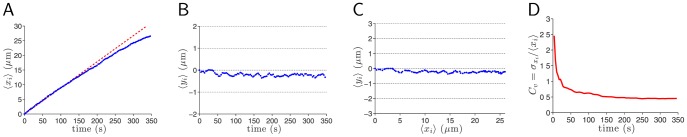
Lagrangian tracking of cells in the 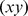-plane of the microchannel. A sample of 205 cells crawling over a plastic hydrophobic surface were subjected to a shear stress 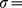
0.18 Pa in the positive 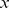-direction, with a soluble calcium concentration of 3 mM. Hundred time samples were collected every 3.5 seconds. (A) Odograph 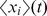 for the average cell position along the signal direction (blue dots). The red dashed line corresponds to a purely uniform translation with 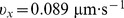. (B) Odograph 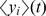 for the average cell position transversely to the signal direction. (C) Average cell trajectory 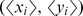. (D) Coefficient of variation 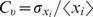 vs. time for the motion along the signal direction.

The average position of the cell along the direction of the shearotactic signal, 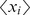, as a function of time (Fig. 5(a)) reveals a behavior typical of a uniform translation for the first 3 minutes of the motion control—the associated constant *x*-component of the velocity, linearly interpolated from our measurements, has a magnitude of 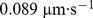, and is represented by the red dashed line in Fig. 5(a). For longer time, 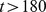 s, we observe a slight slowdown in the motion along the mechanostimulus direction, which, interestingly, does not translate into a speedup along the transverse direction as attested by 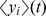 (Fig. 5(b)). This fact is critical to our study as any speedup in the transverse direction ultimately reduces the focus of our “beam” of cells. The average trajectory of the cells further confirms the relatively high focus, on average, of our pack of cells (Fig. 5(c)). It is important adding that the standard deviation of the *x*-position of cells is not small. However, it does not grow over time, relatively to the average position 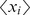, as attested by 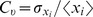 (Fig. 5(d)).

Next, we consider the phase portrait 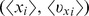 for the motion along the signal direction in the phase space. This phase portrait is obtained using a conditional averaging based upon the average directionality of the 205 cells in our sample. Three increasing threshold levels of directionality were considered giving increasingly higher speed in the 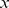-direction (Fig. S2). By comparing Fig. S2(a) and Fig. S2(c), we can confirm that the slowdown in the odograph 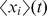, for 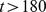 s, is due to cells having a low directionality, thus affecting the overall focus of our beam of cells.

### Mechanotactic Control of Cell Reversal and Trapping

**Figure 6:**
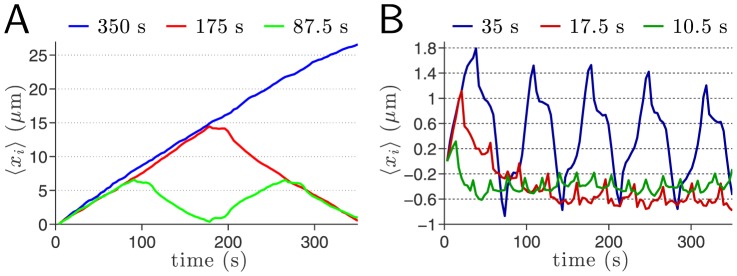
Cell Kinematics: Average displacement along the signal direction for different flow reversal frequencies. Note the difference in scales on the 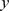-axis for both graphs (Fig. S3). (A) For frequencies smaller than 0.01 Hz, on average the cells clearly undergo a uniform translational migration. When the flow direction is reversed, almost immediately the cells stop moving and it takes approximately 20 s before a motion in the opposite direction is measured. (B) For the frequencies 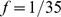 Hz and 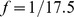 Hz, the cells are still able to follow the faster reversals of signal directions but no longer in a uniform way (i.e. at constant speed). For the even higher frequency 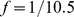 Hz, some changes in the cells' migration direction are discernible but the displacements with respect to the 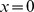 baseline are extremely small: cells are effectively trapped.

Our setup enables us to explore the cellular response to alternating shearotactic signals by reducing the signal switching period—defined as the inverse of the flow reversal frequency—from 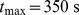 to 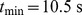. Six different switching periods are considered and for each one of them a minimum of 205 cells and a maximum of 298 cells (Table S2) were tracked in their motion with a sampling time 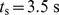 (Fig. 6 and Fig. S3). For the highest switching periods, including 35 s, the cells are clearly able, on average, to reverse course and return to their initial position (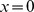) after one or more periods (Fig. 6). For the two smallest switching periods considered, 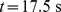 and 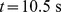 (Fig. 6(b)), cells are still able to respond to the signal reversals albeit much less regularly. The one-dimensional motion along the signal directional ceases to be uniform and the maximum amplitude of the motion in one direction is less than 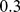 µm—corresponding to approximately 2% of the typical cell body length. We refer to this cell state as effective shearotactic cell trapping, characterized by stalled cell migration and obtained for flow reversal frequencies in the 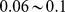 Hz range and above. Note that the definition of chemotactic trapping adopted by Meier et al. [19] corresponds to migration velocities lower than 2 µm/min—higher than the maximum migration velocity of 1.7 µm/min associated with our definition of shearotactic trapping—with random migration angles.

**Figure 7:**
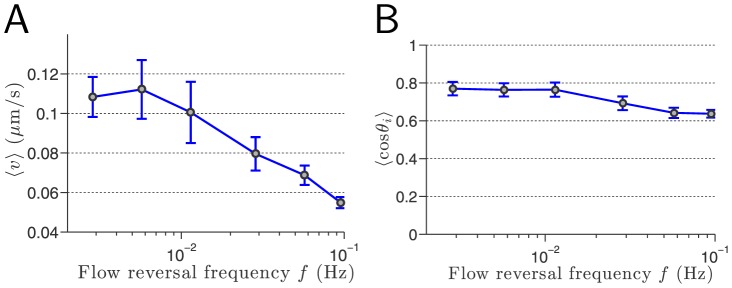
Effects of flow reversal frequency on cellular directionality. (A) Pack average speed 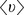 as a function of the flow reversal frequency 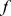 using a log-scale. Beyond the trapping frequency 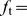
1/17.5 Hz, the pack speed is still significant: 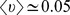 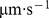 which is to be compared to the average cell speed 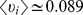 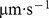 found for the uniform one-dimensional translation along the signal direction. (B): Average 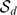 vs. flow reversal frequency. The average directionality does not follow the same trend as the pack speed: it decreases sensibly with 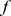. Even when cells are effectively trapped, they still exhibit a fairly high directionality, close to a value of 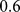.

Given the relatively large size of samples of cells considered (at least 205 cells, Table S2) for each value of the flow reversal frequency, we investigate the pack average speed, 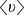, corresponding to the ensemble average over all the cells at all the 100 time samples evenly distributed every 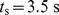. The pack average speed decreases exponentially fast at high flow reversal frequencies (Fig. 7(a)). Even at the trapping frequency 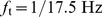, the pack speed is far from being negligible but it essentially corresponds to one “step” forward followed by one “step” backward, thus resulting in the observed effective trapping. This analysis is supported by the variations of the average absolute directionality as a function of the flow reversal frequency (Fig. 7(b)). Indeed, the cell directionality decreases very slightly with the flow reversal frequency. Even at the trapping frequency 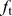, cell still possesses a fairly high directionality which allows it to decide to perform either a “step” forward or a “step” backward.

## Discussion

### Natural Environmental Conditions Provide Optimal Conditions for Shearotactic Guiding

While many groups have been focusing on getting cells to race [22], our main drive is for accuracy, persistence and controllability. This goal is motivated by a growing need for accurate mammalian cell manipulation techniques [12], in relation with biomedical applications in fields like tissue engineering [14], [15] and regenerative medicine [16]. Our results show that the most effective means to achieving persistent directed cellular motion is by mimicking *D. discoideum*'s natural natural environmental conditions in terms of: (i) shear stress levels, and (ii) extracellular soluble calcium levels. Of course, these results cannot be generalized to mammalian cells without extensive additional experiments.

### Effectiveness of Very Low Shear Stress Migration Control

The first critical factor controlling the effectiveness of directed cell motion is the magnitude of 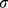, which is intimately related to the intensity of the mechanical signal sensed by the cells [23]. High shear stress values—corresponding to intense shearotactic signals—have been reported to increase both migration speed and directionality [5], [17]. The influence of shear stress on cell speed and directionality for such weak regimes—in the range 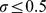 Pa—has never been studied. Understanding the effects of shear stress on cell speed is important but making sure that the cell moves in the right direction—the one we are commanding it to follow using our controlled signaling mechanism—while maintaining adhesiveness to the substrate is paramount given our goal to achieve an effective shearotactic guiding of the cells.

We found that Dd could achieve very high shearotactic efficiency (high 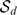 and 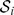) at extremely low shear stress—in the 0.2 Pa range (Fig. 2(b) and Fig. 2(c)). At such shearotactic signaling levels, very few cells detach from the substrate (Table S1). The cell speed does not seem to be very much affected by the low magnitude of the shear stress unless one works with exceedingly low values—below 0.05 Pa (Fig. 2(a)). To the best of our knowledge, these clear shearotactic responses to such extremely low shear stress levels have never been reported before. It is interesting noting that many amoeboid cells dwell in microenvironments where such very low levels of shear stress are common [24]. For instance, interstitial flows, which by nature generate very low shear stress levels, have been shown to affect morphology and migration of melanoma cells [3].

### Optimal Calcium Level for Shearotactic Guiding

The amount of soluble calcium (Ca^2+^) in the extracellular environment is the second factor playing a pivotal role in shearotactic cellular directed guiding and control. Calcium is ubiquitous in the environment of cells which can experience soluble concentrations up to 100 mM [21]. Interestingly, our found optimal level of calcium, 3 mM—when driving vegetative Dd cells on a plastic hydrophobic surface with a shear stress 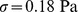 (Fig. 3)—falls into the range of *in vivo* soil conditions, typically between 3.4 and 14 mM [25].

### Extracellular Calcium Level Also Influences Directionality

The extracellular Ca^2+^ has recently been shown to be a key parameter in Dd's motile behavior [21], its chemotactic efficiency [26], as well as its shearotactic prowess—purely in terms of cell speed—and cell-to-surface adhesiveness [17]. Furthermore, Ca^2+^ is a key player in the functioning of stretch-activated mechanosensitive ion channels (SACs) at the root of cellular shear stress sensitivity [27], [28]. Lee et al. [29] first reported that the regulation of cell movement in fish epithelial keratocytes is mediated by SACs. Recently, Lombardi et al. [30] uncovered the existence of calcium-based SACs in Dd cells. However, no noticeable effect on the shearotactic efficiency has been found for [Ca^2+^ ]_ext_ 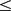
1 mM [17] when considering cells migrating on solid glass surfaces. Fache et al. [17] argue that heterotrimeric G-proteins play a role in the gating of these calcium channels, thereby mediating external calcium entry that eventually stimulates internal calcium release triggering a downstream biochemical cascade affecting cell movement. This process is believed to amplify the mechanical signal and modulate the induced cellular speed [17]. It is also possible that phosphoinositide 3-Kinase (PI3-K), which was found responsible for the directional shearotactic sensing of vegetative Dd cells [5], might be involved at some point of this downstream chain of events.

Contrary to the results reported in [17] where calcium levels only affect speed, our results show that the level of calcium also has an effect on the directionality of vegetative Dd cell motility albeit on plastic surfaces (Fig. 3). Beyond the biological heterogeneity between our experiments and those reported in [17], we believe that the nature of the surface may have some influences on the limits on directional mechanosensing [23]. We speculate that the environmental conditions we used are close to Dd's soil conditions, hence promoting the calcium flux through the SACs and ultimately resulting in improved directional mechanosensing capabilities. The sharp fall in shearotactic efficiency at relatively high calcium concentrations can be explained by a saturation in calcium (Fig. 3).

### Dynamics of Cellular Course Reversal and Trapping

We observe in Fig. 6(a) that for the switching periods 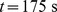 and 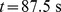, a constant 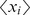 horizontal plateau of approximate duration 20 seconds is clearly visible almost instantly after the signal switches direction (Fig. S3). A similar behavior is also observed for the shorter switching period 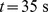 (Fig. 6(b)) albeit less clearly because of the difference in magnification on the *y*-axes in Fig. 6(a) and Fig. 6(b). This lapse of time of approximately 20 seconds seems to be required by the cell to reorganize its internal actin-myosin cytoskeleton as was observed for considerably higher shear stresses [31]. Dalous et al. [31] found that the inversion of cell polarity caused by either mechanical or chemical signals is initiated by the depolymerization of actin at the previous leading edge, which, they found, takes approximately 40 seconds. Furthermore, Dalous et al. [31] proposed the existence of an inhibitory signal that depends on the magnitude of the shear stress and which rapidly spreads from the stimulated edge throughout the entire cell to suppress the polymerization of actin. Our directional control of vegetative Dd cells is achieved at very low shear stress levels, 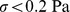, which have never been studied before. According to Dalous et al. [31], the shear stress is too weak—
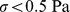
—to trigger an inhibitory signal, which was proposed to suppress the polymerization of actin at the stimulated edge. This faster dynamics we observe possibly indicates a different response mechanism from the actin-myosin reorganization one.

This lag time of 20 seconds—due to the intracellular feedback induced by external mechanostimuli—defines a characteristic frequency 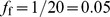 Hz. When the shearotactic signal alternates faster than 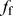, one expects the cell to lack the ability to respond by onset of directed migration. This is exactly what we observe with our found trapping frequency 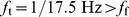: cells fully maintain their directional sensing capabilities but are stalled by the slowness of their motility apparatus.

## Materials and Methods

### Cell growth and preparation

Wild-type D. discoideum AX2 cells (strain obtained from DictyBase; Depositor: Wolfgang Nellen) were grown at 23°C in axenic medium (HL5) on petri dishes [32]. Vegetative cells were harvested during the exponential growth phase with a density not exceeding 1
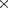
10^6^–4
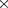
10^6^ cells/mL, pelleted by centrifugation (1000 g, 4 minutes). Cells were then washed twice with MES-Na buffer (20 mM morpholinoethanesulfonic acid, adjusted to pH 6.2 with NaOH) and used immediately. To avoid any damage on cells due to even moderate exposure to light, each sample was used for less than 40 minutes.

### Cell Motility Device Design

Shearotactic cell motility assays were conducted in an optically transparent plastic flow chamber (Fig. 4(a)) in which both magnitude and direction of the shear stress are uniform throughout the 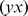-surface of the observation area A located at the center of the channel (Fig. 4(a)), and temporally controlled using an external flow circuit connected to a syringe pump having a highly-controllable flow rate. Vegetative Dd cells (Fig 4(c)) adherent to the bottom surface of the observation area A (Fig. 4(a)) are thereby subjected to an externally-controlled shearotactic signal of very small magnitude. Their migratory responses are tracked by recording the cell trajectories typically over a duration of 20 minutes (Fig. 4(b)) during which some cells travel over 15 times their body length—measured to be on average 14 µm for the cell strain we considered (SI Materials and Methods).

### Experimental setup

Cell tracking experiments were carried out at ambient temperature (23°C) with a Nikon eclipse TE2000-U phase contrast microscope equipped with a 10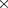 and 20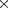 long working distance objectives (SI Materials and Methods). An Apogee Camera (KX32ME) was used to capture the images. We used plastic hydrophobic channel slides (µ-slide VI 0.4 from ibidi) having a width of 3.8 mm, a depth of 0.4 mm, and a length of 17 mm, and a contact angle of 100°. An area of 1 mm^2^ at the center part of each channel was used for measurement.

## Supporting Information

Figure S1
**Influence of shear stress on cell directionality.** (a) The polar histogram demonstrates distribution of angles of net migration vectors for a population of 100 cells; in the absence of any shearotactic signal (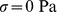) random cellular migration is observed. (b) Shear stress level of 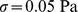 with a population of 126 cells. Even at such exceptionally low shear stress level, we observe a noticeable directional bias in the direction of the shearotactic stimuli given by the red arrow. (c) Shear stress level of 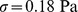 with a population of 135 cells; a large majority of cells are heading in the direction of the mechanostimulus. (d) Shear stress level of 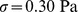 with a population of 129 cells. (e) Shear stress level of 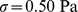 with a population of 73 cells. In all polar histograms, 20 equally-spaced angular bins are considered.(EPS)

Figure S2
**One-dimensional phase portraits corresponding to the signal direction.** A sample of 205 cells crawling over a hydrophobic surface were subjected to a shear stress 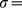
0.18 Pa in the positive 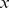-direction, with a soluble calcium concentration of 3 mM. Each phase portrait 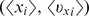 were obtained for different conditional averaging based on the average cellular directionality. (a) 197 cells out of 205 have an average directionality higher than 0.2. (b) 159 cells out of 205 have an average directionality higher than 0.4. (c) 87 cells out of 205 have an average directionality higher than 0.6. The higher the threshold of directionality, the higher the average cell speed along the direction of the signal. Considering even higher level of directionality is not appropriate as the population of cells become too small to yield a reasonable average of 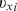. Hundred time samples were collected every 3.5 seconds; slightly less than a quarter of those time samples are shown in the three figures for clarity.(EPS)

Figure S3
**Cell Kinematics: Average displacement along the signal direction for different flow reversal frequencies.** The scale along the *y*-axis is taken identical to the scale used in Fig. 5(b) to allow for an easier comparison of the fine details of the course reversal. (a) Zoom-in for the only reversal corresponding to the switching period 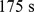; (b) Zoom-in for the first reversal corresponding to the switching period 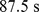; (c) Zoom-in for the second reversal corresponding to the switching period 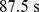.(EPS)

Figure S4
**Prescripted cellular course.** Left column: (Movie S2) right, trapped and left. Right column: (Movie S3) right, trapped, left and right. For each column: (a) Desired cellular output; (b) Imposed externally controlled mechanostimulus; (c) Measured cellular displacement in the 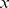 direction; (d) Instantaneous snapshot of the cell in the observation area at instant 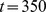 s. The red arrow indicates the mechanostimulus direction. The extracellular calcium concentration is 3 mM.(EPS)

Figure S5
**Schematic of cell kinematic parameters.** The cell position, corresponding to its centroid position, is represented as successive points at instants 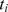 and 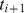 in Cartesian coordinates. In all our experiments, the shearotactic signal is aligned with the *x*-axis but its direction is given either by the positive or the negative *x*-direction.(EPS)

Table S1
**Detachment rate (DR) for unpolarized vegetative Dd cells crawling on a plastic hydrophobic surface over a time span of 1200 seconds for different shear stress levels and different extracellular calcium concentrations.** Each entry is calculated using populations comprising between 200 to 300 cells with an estimated precision of the order of one percent. Even for mild values of the shear stress—with respect to typical levels considered in earlier studies [5], [17], [18]—the detachment rate is significant: in the 50% range for 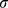 = 0.5 Pa. Even weaker shear stress levels, in the 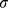 = 0.18 Pa range, yield minimal detachment rates, between 1 to 3%, for a broad range of extracellular calcium concentrations. Such low levels of detachment rates are very close to the ‘natural’ detachment rate measured in the absence of any flow.(EPS)

Table S2
**Cell populations considered for the one-dimensional cell motion control, cell reversal and cell trapping experiments.** The Lagrangian path of each individual cell is tracked in the observation area using a reduced sampling time, 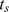, of 3.5 seconds which was found appropriate given the average cell speed of approximately 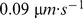. The smallest population size comprises 205 cells, which is sufficient to yield statistically relevant data.(EPS)

Movie S1 **Prescripted cellular courses were sought corresponding to the following sequence of motion: right, trapped, right, left (** **Fig. 1** **).**
(MP4)

Movie S2 **Prescripted cellular courses were sought corresponding to the following sequence of motion: right, trapped, left (Fig. S4 left column).**
(MP4)

Movie S3 **Prescripted cellular courses were sought corresponding to the following sequence of motion: right, trapped, left, right (Fig. S4 right column).**
(MP4)

File S1 **Supporting text.**
(PDF)
