# Supplementary material for: Persistent Cellular Motion Control and Trapping Using Mechanotactic Signaling
Source: PLoS One. 2014 Sep 10;9(9):e105406. doi: 10.1371/journal.pone.0105406 (PMC4160188; doi:10.1371/journal.pone.0105406)
Supplement: File S1 — Supporting text. (PDF) [file pone.0105406.s011.pdf]

Supplementary Information: Persistent cellular motion control and trapping using mechanotactic signaling

Xiaoying Zhu[1,2], Roland Bouffanais[1,2] and Dick K. P. Yue[2]

[1]*Singapore University of Technology and Design, 20 Dover Drive, Singapore 138682,* [2]*Department of Mechanical Engineering, Massachusetts Institute of Technology, Cambridge, MA 02139, USA*

I. SI TEXT

A. Influence of Shear Stress on Cell Directionality

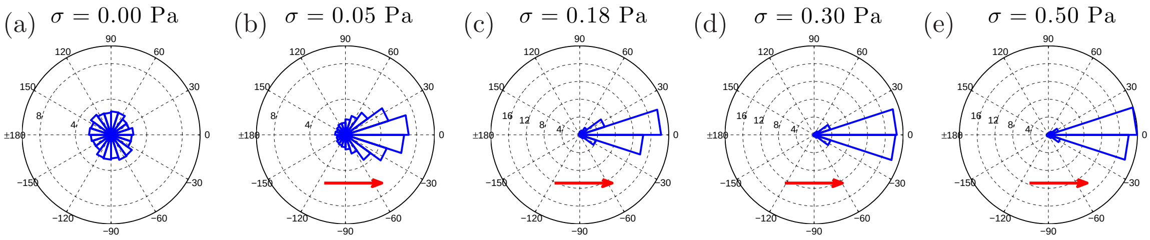

FIG. S1. Influence of shear stress on cell directionality. (a) The polar histogram demonstrates distribution of angles of net migration vectors for a population of 100 cells; in the absence of any shearotactic signal ($\sigma = 0$ Pa) random cellular migration is observed. (b) Shear stress level of $\sigma = 0.05$ Pa with a population of 126 cells. Even at such exceptionally low shear stress level, we observe a noticeable directional bias in the direction of the shearotactic stimuli given by the red arrow. (c) Shear stress level of $\sigma = 0.18$ Pa with a population of 135 cells; a large majority of cells are heading in the direction of the mechanostimulus. (d) Shear stress level of $\sigma = 0.30$ Pa with a population of 129 cells. (e) Shear stress level of $\sigma = 0.50$ Pa with a population of 73 cells. In all polar histograms, 20 equally-spaced angular bins are considered.

σ (Pa)	0.05	0.18	0.3	0.5
DR($[Ca^{2+}]_{ext}$=3 mM)	1%	3%	5%	50%

$[Ca^{2+}]_{ext}$ (mM)	0.01	0.1	1	3	5	10	50
DR(σ=0.18 Pa)	1%	1%	2%	3%	1%	2%	1%

TABLE S1. *Detachment rate (DR) for unpolarized vegetative Dd cells crawling on a plastic hydrophobic surface over a time span of 1200 seconds for different shear stress levels and different extracellular calcium concentrations. Each entry is calculated using populations comprising between 200 to 300 cells with an estimated precision of the order of one percent. Even for mild values of the shear stress—with respect to typical levels considered in earlier studies [1–3]—the detachment rate is significant: in the 50% range for σ=0.5 Pa. Even weaker shear stress levels, in the σ=0.18 Pa range, yield minimal detachment rates, between 1 to 3%, for a broad range of extracellular calcium concentrations. Such low levels of detachment rates are very close to the 'natural' detachment rate measured in the absence of any flow.*

Switching period (s)	Number of cells tracked
10.5	298
17.5	283
35	294
87.5	222
175	269
350	205

TABLE S2. *Cell populations considered for the one-dimensional cell motion control, cell reversal and cell trapping experiments. The Lagrangian path of each individual cell is tracked in the observation area using a reduced sampling time,* t_s*, of 3.5 seconds which was found appropriate given the average cell speed of approximately* 0.09 $\mu m \cdot s^{-1}$*. The smallest population size comprises 205 cells, which is sufficient to yield statistically relevant data*

B. One-dimensional Cell Motion Control

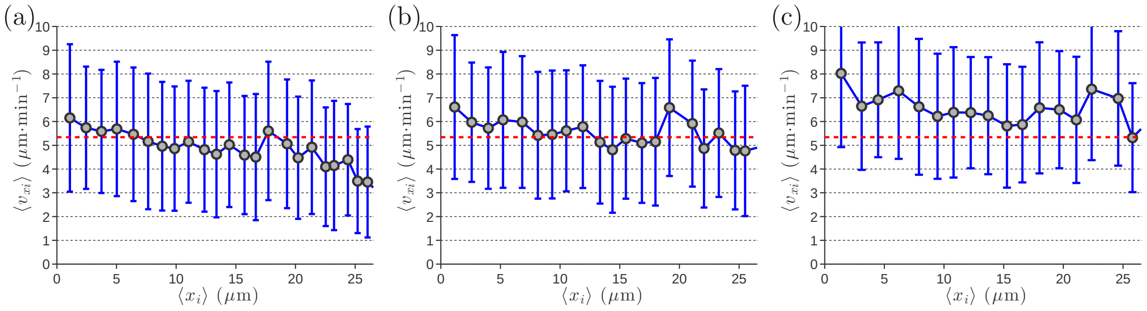

FIG. S2. One-dimensional phase portraits corresponding to the signal direction. A sample of 205 cells crawling over a hydrophobic surface were subjected to a shear stress σ =0.18 Pa in the positive x-direction, with a soluble calcium concentration of 3 mM. Each phase portrait $(\langle x_i \rangle, \langle v_{xi} \rangle)$ were obtained for different conditional averaging based on the average cellular directionality. (a) 197 cells out of 205 have an average directionality higher than 0.2. (b) 159 cells out of 205 have an average directionality higher than 0.4. (c) 87 cells out of 205 have an average directionality higher than 0.6. The higher the threshold of directionality, the higher the average cell speed along the direction of the signal. Considering even higher level of directionality is not appropriate as the population of cells become too small to yield a reasonable average of v_{xi}. Hundred time samples were collected every 3.5 seconds; slightly less than a quarter of those time samples are shown in the three figures for clarity.

C. Mechanotactic Control of Cell Motion, Reversal and Trapping

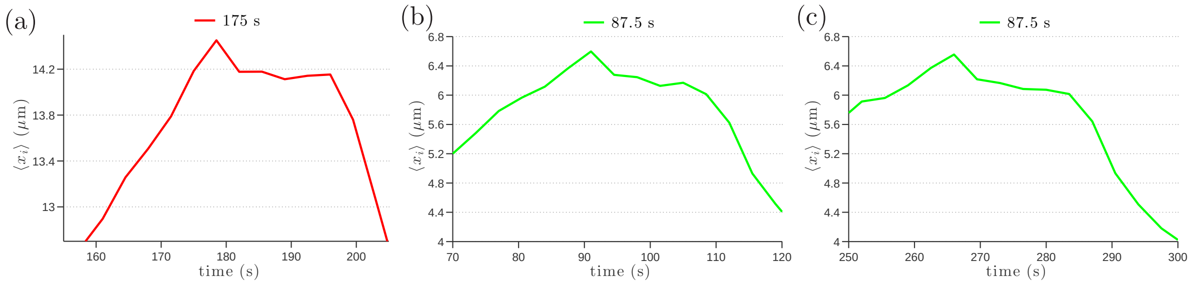

FIG. S3. Cell Kinematics: Average displacement along the signal direction for different flow reversal frequencies. The scale along the y-axis is taken identical to the scale used in Fig. 5(b) to allow for an easier comparison of the fine details of the course reversal. (a) Zoom-in for the only reversal corresponding to the switching period 175 s; (b) Zoom-in for the first reversal corresponding to the switching period 87.5 s; (c) Zoom-in for the second reversal corresponding to the switching period 87.5 s .

D. Prescripted Cellular Courses

Three particular prescripted cellular courses were sought corresponding to the following sequences of motion:

(1) right, trapped, right, left (Fig. 7): Movie S1

(2) right, trapped, left (Fig. S4 left column): Movie S2

(3) right, trapped, left, right (Fig. S4 right column): Movie S3

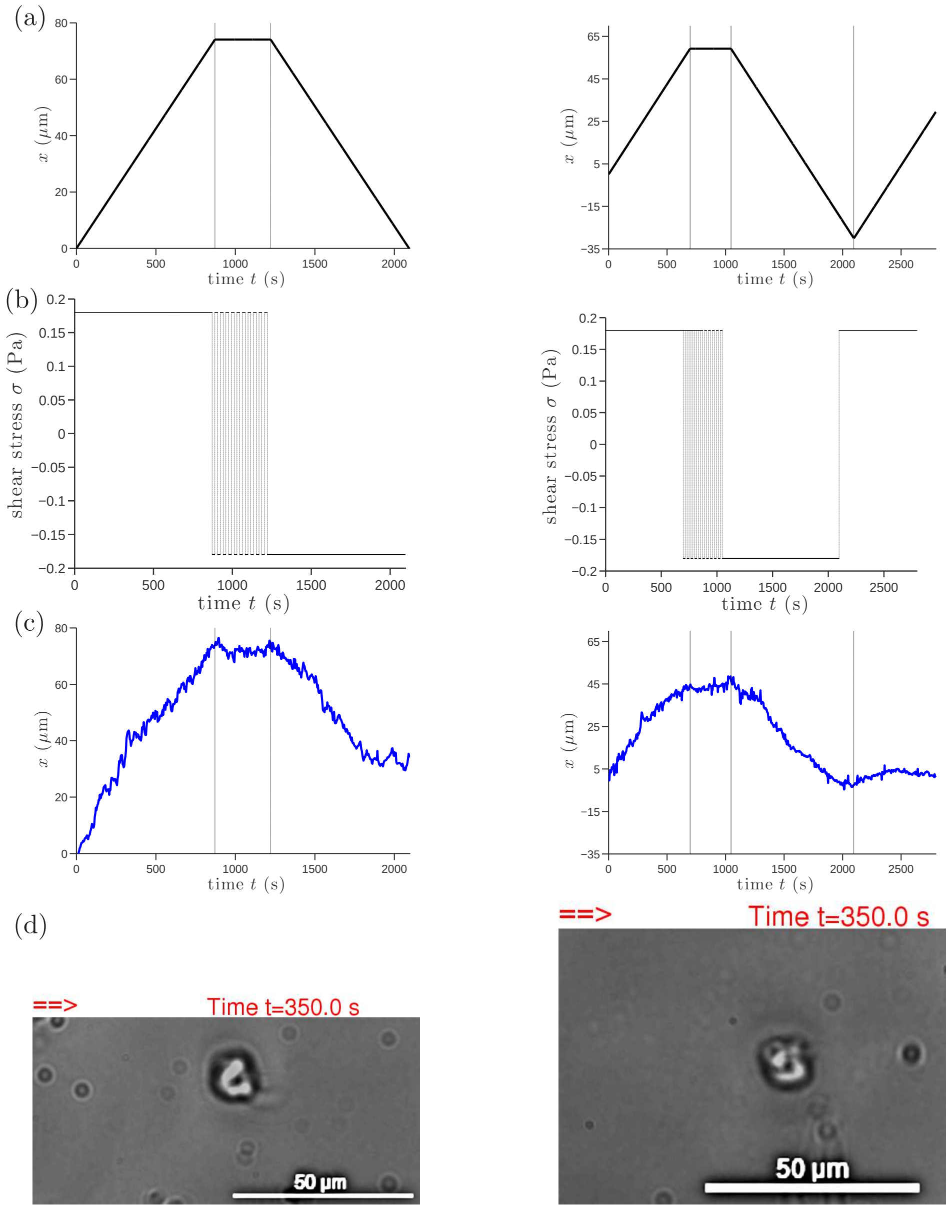

FIG. S4. Prescripted cellular course. Left column: (Movie S2) right, trapped and left. Right column: (Movie S3) right, trapped, left and right. For each column: (a) Desired cellular output; (b) Imposed externally controlled mechanostimulus; (c) Measured cellular displacement in the x direction; (d) Instantaneous snapshot of the cell in the observation area at instant $t = 350$ s. The red arrow indicates the mechanostimulus direction. The extracellular calcium concentration is 3 mM.

II. SI MATERIALS AND METHODS

A. Experimental setup

A dual-rate syringe pump (KDS) was used to generate continuous creeping flows with a flow rate ranging from 0.1 mL/min to 10 mL/min. After being washed twice with the MES-Na buffer, cells were resuspended in according buffers at a density of 10^5–10^6 cells/mL. Resuspended cells were immediately introduced into the channel slide and allowed to settle for 10 minutes. The surface density was approximately 50 cells/mm^2, corresponding to a less than 1 % surface coverage. No hydrodynamic interaction between adjacent cells was detected during the experiments.

The action of shear flow on the cell adhering to the substrate is determined by the hydrodynamic forces exerted on the cell. When the Reynolds number is small, inertial effects can be neglected. In a laminar flow, the net force and torque exerted on an adhering cell, considering it as an elastic solid, are proportional to the wall shear stress. In the geometry of the µ-Slide VI 0.4 channel (from ibidi), the shear stress is given by

$$\sigma = \frac{6\eta D}{wh^2}, \quad (1)$$

where D is the flow rate, η the dynamic viscosity of the fluid, w the width of the channel, and h the height of the channel. The critical shear stress for cell detachment $\sigma_{1/2}$—for which 50 % of cells are detached—is 0.5 Pa for D. discoideum cells on plastic hydrophobic channels (ibidi) over 20 minutes time. In order to accurately measure the persistent directed shearotactic migration, cells peeled away from the substrate during both the 20-min or 6-min measurements were excluded. As shown in Table S1, this peeling process is marginal at very low shear stress levels.

B. Data acquisition

A sequence of 80 (resp. 100) images was captured every 15 (resp. 3.5) seconds to track cell motility leading to a complete exposure to signal of 20 (resp. 6) minutes. The reduced frame rate of (3.5 s)$^{-1}$ is used to capture fine kinematic details of cell reversal and trapping when swiftly reversing the mechanostimulus direction. The first image was taken after the flow was applied for 30 seconds. Images were analyzed with Image-Pro Premier 9.0 software (MediaCybernetics, MD, USA). The objects in each frame were segmented and their contours were detected. A new image with a binary mask of the segmented objects was created. The centroid of each object is then calculated on the center of mass of the binary mask of the object. This analysis procedure was repeated for all images and data was exported in Matlab (© The MathWorks Inc.). It should be noted that individual cells differ from each other primarily because of the difference in the contact zone to surfaces, hence showing different levels of roundness and diameters. Because we measured a large number of cells (approximately 100 cells at the (15 s)$^{-1}$ frame rate—Fig. S1—and over 200 at the (3.5 s)$^{-1}$—Table S2), such details of individual cells were averaged. The average diameter of cells was determined to be 14 µm.

C. Analysis of cell kinematics

The centroid positions, (x_i, y_i), of individual cells in the (xy)-bottom plane of the channel slide were extracted from the series of 80 to 100 images depending on the frame rate chosen. The instantaneous velocity, (v_{xi}, v_{yi}), is obtained through first-order finite differences. The instantaneous velocity along the x-axis is calculated from the following formula:

$$v_x(t_i) = \frac{x(t_{i+1}) - x(t_i)}{t_{i+1} - t_i}, \quad (2)$$

where $x(t_i)$ denotes the cell position in the i-th frame at time t_i along the x-axis, and $v_x(t_i)$ denotes the velocity component, at instant t_i, in the direction of the x-axis, which is systematically taken parallel to the mechanostimulus direction. The instantaneous velocity component along the y-direction is computed in a similar way by simply replacing x by y. The instantaneous angle θ_i is defined as the angle between the velocity vector and the horizontal axis (x-axis), classically measured in counterclockwise direction (see Fig. S5). Practically, this process—simply amounting to a first-order time derivative—for obtaining the velocity and speed of cells is known to amplify the noise and fluctuations in the cell position. Consequently, the velocity was obtained with an 11-point Savitzky and Golay differentiation filter

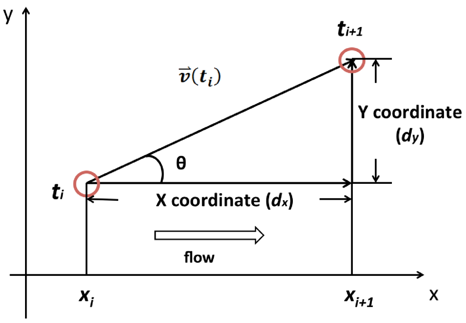

FIG. S5. Schematic of cell kinematic parameters. The cell position, corresponding to its centroid position, is represented as successive points at instants t_i and t_{i+1} in Cartesian coordinates. In all our experiments, the shearotactic signal is aligned with the x-axis but its direction is given either by the positive or the negative x-direction.

effectively smoothing the high-frequency fluctuations. The application of this differentiation filter was found to have very negligible effect on the average velocity components and average speed.

---

[1] Décavé E, Rieu D, Dalous J, Fache S, Bréchet Y, et al. (2003) Shear flow-induced motility of *Dictyostelium discoideum* cells on solid substrate. J Cell Sci 116: 4331–4343.
[2] Fache S, Dalous J, Engelund M, Hansen C, Chamaraux F, et al. (2005) Calcium mobilization stimulates *dictyostelium discoideum* shear-flow-induced cell motility. J Cell Sci 118: 3445–3457.
[3] Décavé E, Garrivier D, Bréchet Y, Fourcade B, Brückert F (2002) Shear flow-induced detachment kinetics of *dictyostelium discoideum* cells from solid substrate. Biophys J 82: 2383–2395.
